# Ultrafast Surface Plasmon Probing of Interband and Intraband Hot Electron Excitations

**DOI:** 10.1021/acs.nanolett.4c01669

**Published:** 2024-06-04

**Authors:** Péter Sándor, Béla Lovász, Judit Budai, Zsuzsanna Pápa, Péter Dombi

**Affiliations:** † HUN-REN Wigner Research Centre for Physics, 1121 Budapest, Hungary; ‡ ELI-ALPS Research Institute, 6728 Szeged, Hungary

**Keywords:** ultrafast phenomena, hot electrons, interband, intraband, plasmon, pump−probe

## Abstract

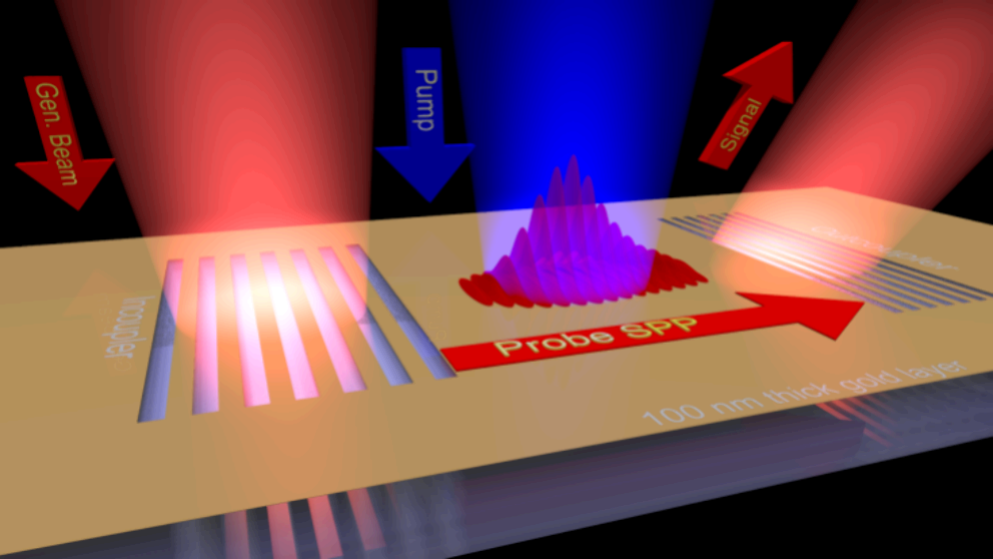

Upon the interaction of light with metals, nonthermal electrons are generated with intriguing transient behavior. Here, we present femtosecond hot electron probing in a noveloptical pump/plasmon probe scheme. With this, we probed ultrafast interband and intraband dynamics with 15 nm interface selectivity, observing a two-component-decay of hot electron populations. Results are in good agreement with a three-temperature model of the metal; thus, we could attribute the fast (∼100 fs) decay to the thermalization of hot electrons and the slow (picosecond) decay to electron–lattice thermalization. Moreover, we could modulate the transmission of our plasmonic channel with ∼40% depth, hinting at the possibility of ultrafast information processing applications with plasmonic signals.

Light-induced interband and intraband excitations in various semiconductor media, metals, interfaces and nanosystems have found important real-life applications recently. These include high-harmonic generation in solids,^1−3^ hot electron-enhanced photochemistry^4−6^ (e.g., photocatalysis of water splitting^7^) and sensing.^8,9^ In combination with surface plasmon polariton (SPP) generation,^10^ these excitations even enabled the construction of ultrafast, active plasmonic switches and nanoscale optical circuitry,^8,11−13^ paving the way toward surface-integrated nanooptical devices with a switching speed surpassing that of state-of-the-art microelectronics by at least 4 orders of magnitude.^14^ With these prospects in mind, it is of great importance that fundamental physics of light-induced interband and intraband processes is (i) resolved in time on an ultrafast time scale and (ii) based on this knowledge, pathways toward full light-field control of electron motion in solids are explored.

Fundamental research on nonthermal and hot electron generation and decay revealed important aspects on how light interacts with the electron subsystem and what the main decay mechanisms are in different media. Electron distributions are mainly determined by thermalization in the case of continuous excitation, found by Dubi et al.^15^ Multiple experimental and theoretical studies have revealed the energy distribution of nonthermal electrons and the electronic system around the Fermi level in the case when SPPs are generated with ultrashort laser pulses.^16−19^ Khurgin identified the main absorption mechanisms in metals and pointed out their efficiency in hot carrier generation.^20^

Because nonthermal electron processes are typically exploited at interfaces, one needs a suitable probing tool that has ultrahigh interface selectivity. On the other hand, the ultrafast nature of both the excitation and the decay process prompt for an ultrafast probe. Transient reflectivity measurements on various model systems already enabled intraband excitation investigations. The early transient signal is deeply influenced by the nonthermal character of the electron distribution perturbed by a double steplike function extending up to the photon energy of the excitation around the Fermi energy.^21,22^ Electron excitation can be more efficient with a configuration supporting plasmon excitation as shown in a comparative study of Heilpern et al. on photon absorption processes with and without SPP excitation via time-resolved reflectometry.^17^ Considering interband absorption processes in gold, most of the energy is consumed by promoting the electron from the d-shell to the Fermi level^23^ limiting the accessible energy levels to the difference between the pump photon energy and the interband transition threshold (1.8 eV).^24^

Due to the wave nature of light, the diffraction limit and field penetration depths set a boundary on how selectively nonthermal electron processes can be probed at interfaces. Here, we employ a noveloptical pump/SPP probe approach to investigate ultrafast dynamics of both interband and intraband excitations at a gold-air interface. An SPP probe has several advantages over conventional optical methods; (i) it offers very high interface selectivity of some 15 nm due to the extremely short decay lengths of plasmonic fields,^25,26^ and, more importantly, it holds the promise of probing nanometric volumes when combined with tailored surface waveguides; (ii) it also offers ultrahigh temporal resolution in a pump–probe scenario due to the possibility of generating few-cycle plasmonic wavepackets.^27−29^ This way, we could demonstrate the realization of nonthermal electron population probing with ultrahigh spatiotemporal resolution in an all-optical scenario. With this tool at hand, we could investigate intraband and interband processes with ultrahigh surface selectivity. Our experimental observations are well reproduced with an extended three-temperature model (3TM), allowing us to track hot electron population decay mechanisms taking place at closest nanometric vicinity of the gold-air interface.

For investigating nonthermal intraband and interband excitations, we need light pulses with different colors (see Figures 1(a) and 1(b)). To realize this, first we directly illuminate a 100 nm thin gold film either with near-infrared or with blue pump pulses at a repetition rate of 1 kHz using s-polarization in both cases. Intraband transitions are excited by 800 nm laser light directly from a Ti:sapphire amplifier with 38 fs pulse length. Interband transitions are induced by 480 nm laser pulses from a femtosecond optical parametric amplifier with 96-fs pulses. In order to realize an ultrahigh selectivity interface probe with sufficient temporal resolution, we generated SPPs on the gold film by coupling temporally synchronized 800 nm pulses to the interaction region using an SPP grating coupler structure (incoupler) milled into the gold film deposited on a fused silica substrate, as shown in Figure 1(c). The gold layer was produced with thermal evaporation and its root-mean-square roughness was measured to be 0.6 nm. The plasmon propagates about 30 μm (which is much shorter than the SPP decay length) until it encounters a second grating structure (outcoupler) which turns it to a pulse propagating in free space again, the spectrum of which is detected using a spectrometer. We note that the same sample was used in case of both pump wavelengths. We also checked that the plasmonic wavepacket inherits the temporal shape of the probe pulses (measured before hitting the incoupling grating structure) and thus, 38 fs probing is possible with our setup (see Supporting Information, Sec. I on the details of the measurement scheme and the plasmonic wavepackets’ temporal shape).

**Figure 1:**
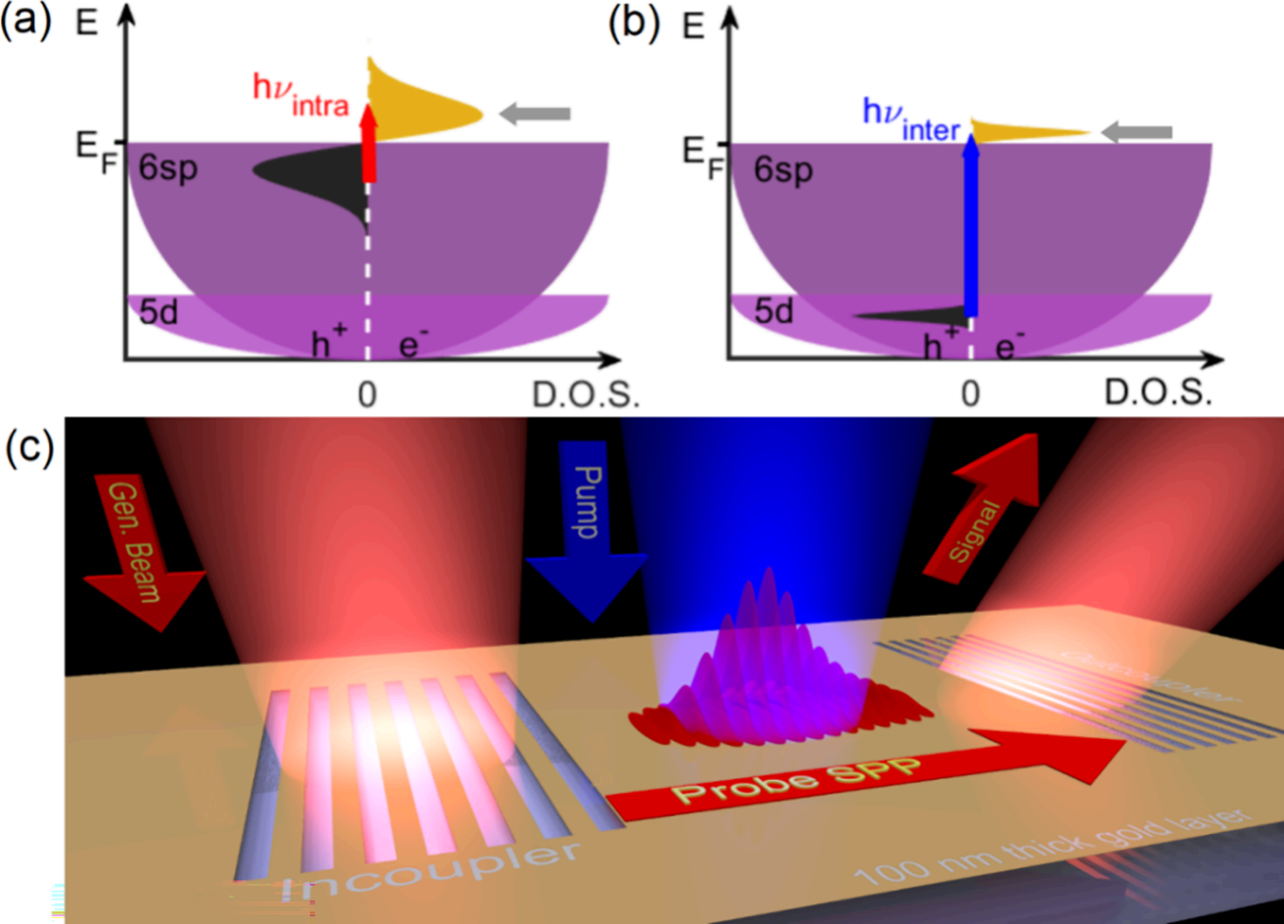
Concept and arrangement for the experiment. (a, b) Schematic diagram of generating nonthermal electrons and holes in gold using intra- and interband optical excitations, respectively. (c) Illustration of the pump–probe concept with the generation and outcoupling of SPPs using grating couplers milled into the 100 nm gold layer. Nonthermal electron distribution is generated in between the grating couplers by a separate, time-delayed pump pulse (shown in blue color).

Figures 2 and 3 show measured pump–probe data for intraband and interband excitations, respectively. For both Figures, the normalized power throughput is plotted as a function of delay in panel (a) for a number of pump peak intensities (quoted in the legend in units of TW/cm^2^; lighter color shades/red or blue, respectively/represent higher intensities). The *x*-axis is linear between −1 and 5 ps, and logarithmic above 5 ps to show the data for the full range of time delays. The curves with different intensities are shifted along the *y*-axis for a clear presentation. The curves in Figures 2(a) and 3(a) are the result of averaging 5 and 10 repetitions, respectively, and are also smoothed by calculating a running average that involves two neighboring data points on each side.

**Figure 2:**
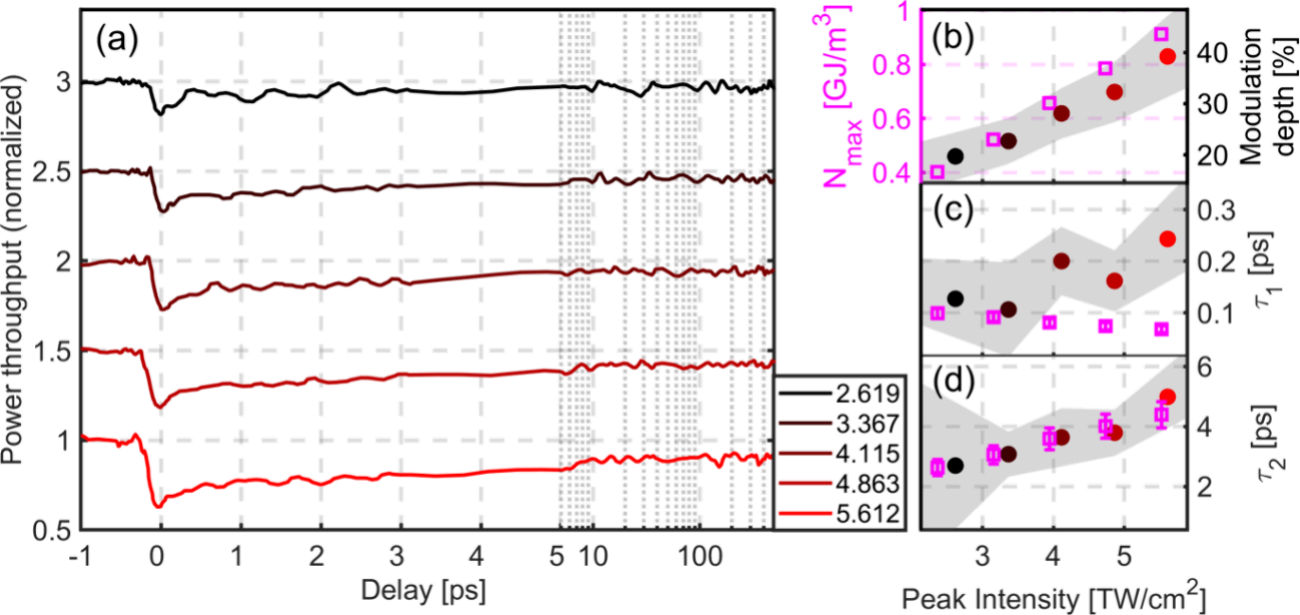
Relaxation of ultrafast intraband excitations (800 nm pump) at a plasmonic interface. (a) Light throughput as a function of pulse delay, for a set of different pump peak intensities (in TW/cm^2^). (b) Modulation depth as a function of pump peak intensity (red-shaded solid dots) and calculated maximal energy density stored in the nonthermal electrons (magenta squares). (c, d) Fitted (red-shaded solid dots) and calculated (magenta squares) τ_1_ and τ_2_ parameters, respectively. Grayscale shading signifies the uncertainty (the full width at a given data point corresponds to two standard deviations) of the experimental data.

**Figure 3:**
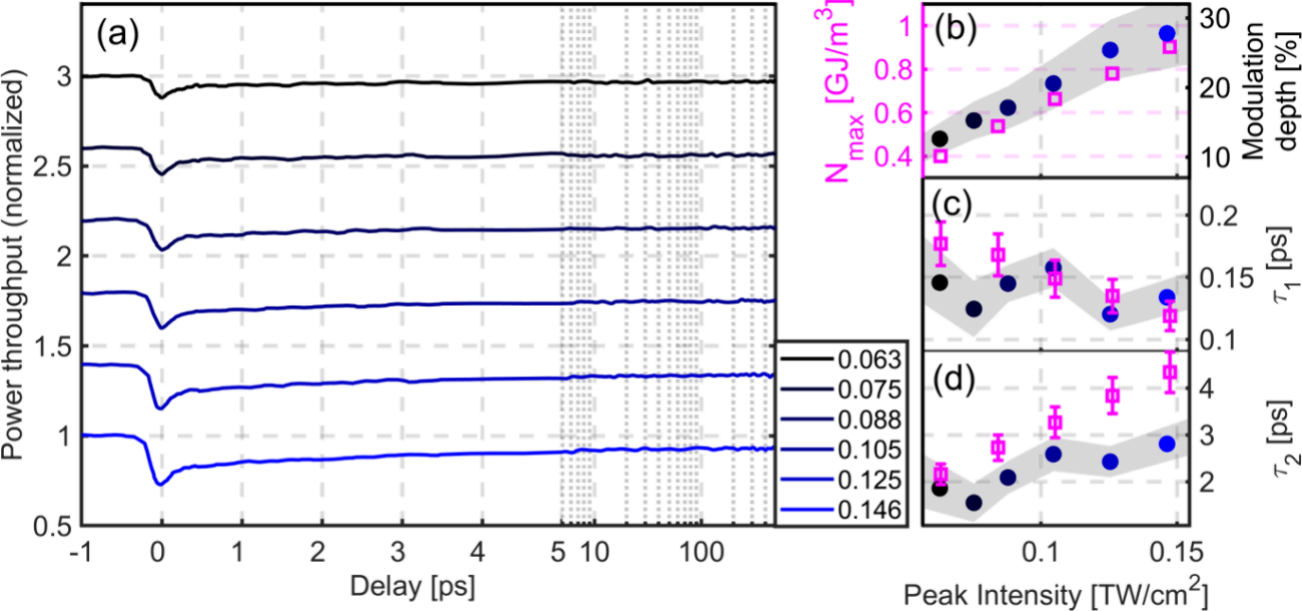
Relaxation of ultrafast interband excitations (480 nm pump) at a plasmonic interface. (a) Light throughput as a function of pulse delay, for a set of different pump peak intensities (in TW/cm^2^). (b) Modulation depth as a function of pump peak intensity (blue-shaded solid dots) and calculated maximal energy density stored in the nonthermal electrons (magenta squares). (c, d) Fitted (blue-shaded solid dots) and calculated (magenta squares) τ_1_ and τ_2_ parameters, respectively. Grayscale shading signifies the uncertainty (the full width at a given data point corresponds to two standard deviations) of the experimental data.

At negative delays, the throughput is fluctuating around unity, as expected. Close to zero delay, with the arrival of the pump pulse, the signal falls sharply due to gold entering a strongly nonequilibrium state that is less conducive to plasmon propagation. This nonequilibrium state is characterized by the appearance of energetic, nonthermal electrons around the cold ion cores.^30^ Once the pump pulse is gone, the system starts to relax on three different time scales. Nonthermal electrons thermalize with the rest of the electron subsystem in a few tens to a few hundreds of femtoseconds through electron–electron collisions.^15,22^ The electrons are also coupled to the lattice, and energy is transferred to the latter in a few picoseconds. These two time scales can be clearly distinguished in our data, especially in Figure 3(a) at higher intensities. The fact that cooling of the lattice itself is not concluded by the end of a given scan (at 500 ps) is also evident from the data, because at that point the light throughput still stays significantly below unity. Hence, the time scale associated with this process is presumably much longer than 1 ns.

The clear separation of the time scales allows us to characterize the dynamics and fit the data with a function that is the combination of two exponentials and a linear term, as given by , where τ_1_ and τ_2_ are time constants of nonthermal electron relaxation and electron–lattice thermalization, respectively. Since lattice relaxation takes much longer than what our pulse delay range allows us to see, we approximated this contribution to the data with a linear function. For both Figures 2 and 3, panels (c) and (d) show the fitted τ_1_ and τ_2_ relaxation constants, respectively, for different peak intensities. In panel (b) the modulation depth is plotted, which we define as  where *S*_*min*_ is the minimum value of the light throughput (around zero delay) and *S*_0_ is its value at negative delays. Basically the τ_2_ relaxation constant and the modulation depth values exhibit an increasing tendency with increasing intensity, while for the τ_1_ values more pronounced differences can be obtained for the two wavelengths.

We measured the τ_1_ parameter to be between 100 and 250 fs for both the intraband and interband excitations (Figure 2(c) and 3(c)). An important difference is that while the τ_1_ values belonging to the intraband excitation increase slightly with increasing intensities, the tendency is quite different for the interband case. The relatively large uncertainties in τ_1_ can be attributed to the low number of data points that are available for fitting it. Furthermore, in case of the 800 nm pump, there is some leakage of it into the detector, and since it spectrally overlaps with the probe, it increases the noise level. Values for the τ_2_ parameters are between 2 and 5 ps (Figure 2(d)) and between 1 and 4 ps (Figure 3(d)). The order of magnitude of these values is consistent with earlier reports in the literature.^21,31−33^

Figures 2(b) and 3(b) show the modulation depths. A key difference between experiments using different pump wavelengths is the fact that at 480 nm, much lower peak intensity was needed to achieve the same modulation depth in the plasmonic probe channel. We note that the maximum pump intensity at each wavelength was chosen to avoid sample damage. Before and after each measurement, the sample was directly visualized for intactness using a compact digital microscope, and no damage was observed for the data shown.

Different pump wavelengths excite different transitions. When the photon energy is lower than the interband optical transition threshold (∼1.8 eV in gold), intraband absorption processes take place. This is the case for the 800 nm pump wavelength (1.55 eV). On the contrary, for 480 nm (2.58 eV), interband absorption processes become dominant, during which electrons are excited from the d-band to the sp band. These transitions change the internal energy dynamics of gold, which can be described by 3TM. The partial differential equations contain the following variables: the energy density stored in the nonthermal electrons (*N*), the temperature of the thermalized electrons (*T*_*e*_) and the temperature of the lattice (*T*_*l*_)^34^ (see Section II of the Supporting Information for more details on the 3TM).

The 3TM model enables us to distinguish between the different pump wavelengths by taking into account the different reflection and absorption properties of gold. Furthermore, the energy dependent lifetime of the nonthermal electrons is also introduced by the parameter *a*, which implies that the electron–electron collision rate is electron energy dependent, as given by the Fermi liquid theory,^15^ and can be calculated as *a* = τ_*ee*_^–1^ = *K*[(*πk*_*B*_*T*_*e*_)^2^ + (*E* – *E*_*F*_)^2^], with  providing the best correspondence with our results, being consistent with literature.^15,35,36^ To set the *a* parameter properly, the achievable energy levels of electrons after photon absorption have to be considered. For intraband excitations, after photon absorption, electrons are promoted to above the Fermi level, and the electron occupation probability is perturbed by an amount that is commonly assumed to be a double steplike function extending up to the photon energy of the excitation around Fermi energy (±1.55 eV in our case).^22^ However, for the interband excitations, most of the energy is consumed by overcoming the 1.8 eV band edge. This means that although the energy of the incoming photons is larger in this case, the photoexcited electrons are distributed near the Fermi level. Furthermore, the initially assumed uniform distribution quickly rearranges itself in such a way that most energetic electrons move to an energy level near the center of the distribution.^22^ Based on this, in our simulations we considered that the bulk of the detected signal is provided by electrons having 0.8 eV excess energy for intraband excitation, and 0.4 eV for interband excitation. By setting the model parameters accordingly, our 3TM calculations provide the changes in *N*, *T*_*e*_ and *T*_*l*_ as a function of time. Since the SPP travels at the gold-air interface, its propagation will be affected by the changes induced on the top of the layer (*N* and *T*_*e*_ values obtained at the top of the gold layer for different applied excitation fluences are plotted in Figure 4).

**Figure 4:**
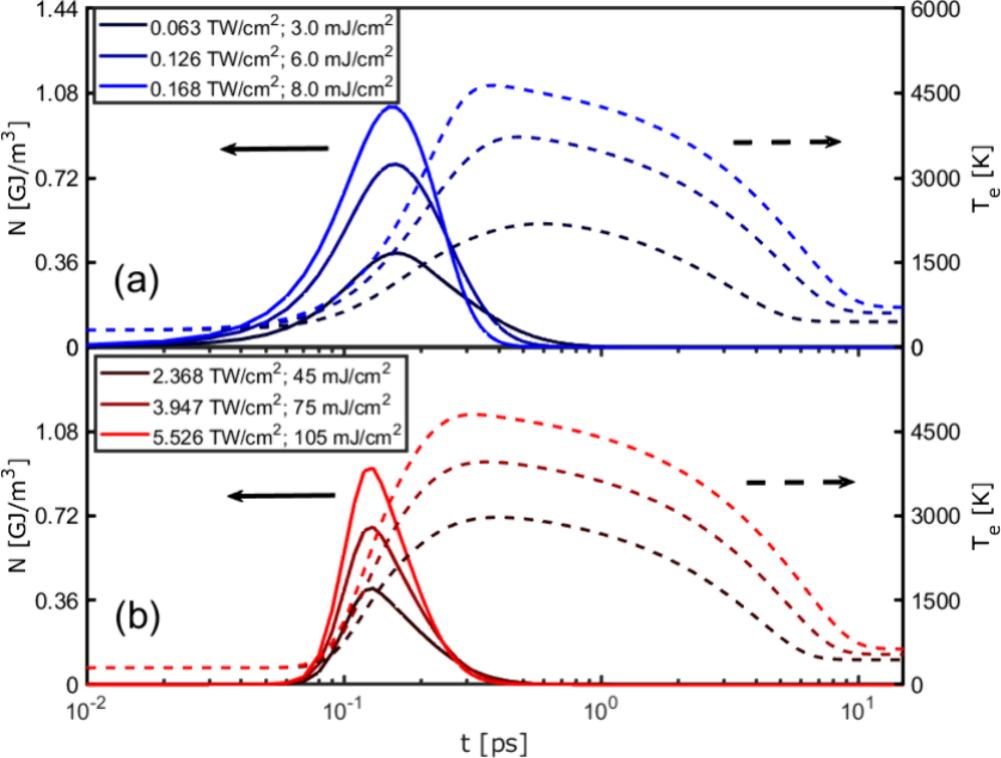
(a) *N* (solid lines) and *T*_*e*_ (dashed lines) for 480 nm pump as a function of time for different peak intensities (fluences), monitored on the top of the gold layer. (b) same as in (a), but for 800 nm pump. Arrows point to the respective vertical axes. (Note that the horizontal axis is logarithmic.)

By having these dynamics we can interpret the origin of the experimental observations. Let us consider first the origin of the signal drop of SPP transmission. According to the literature,^30,34^ elevated electron temperatures increase the imaginary part of the dielectric function for the wavelength of our plasmon probe. This change originates from the modification of the electron distribution. While for nonthermal electrons the electron distribution exhibits the already mentioned doubled step-like shape, the main character of the distribution is similar to the one belonging to an elevated electron temperature, in the sense that both mean an increase above/decrease below the Fermi-level. Therefore, the two different cases will result in an increased imaginary part of the dielectric function. As such increase corresponds to larger losses, it results in larger absorption of the SPP probe. Taking into account the different birth time of the nonthermal and thermal electrons, the sharpest peak in the experimentally observable transmission drop can be assigned to nonthermal electrons, while the second drop observed hints at the appearance of a thermalized electron population. The role of nonthermal electrons is also supported by the intensity dependence of *N*, which follows the experimentally observed intensity dependence of the modulation depth (c. f. Figure 2 and 3).

Regarding the short decay, there are several aspects to analyze. Looking at the 480 nm pump wavelength, quantitative comparison of the experimental τ_1_ parameter with the τ_1_ from the model shows that model values exhibit a decreasing tendency, starting from around 200 fs for the lowest intensity, and mostly running together with the experimental values within the error limit. The decrease is the consequence of the behavior of electron–electron scattering time. For 480 nm pump wavelength, the excess energy of the electrons is supposed to be small as they have to overcome the interband threshold first. This means that their electron–electron scattering time is in the few hundred fs range, but at the same time, it drops immediately as the excess energy is transferred to the electron subsystem increasing its temperature (see Figure S4 (c)). By increasing the excitation intensity, the electron system is heated to higher temperatures (dashed lines in Figure 4(a)) and the corresponding decay time of the nonthermal electrons gets shorter (solid lines in Figure 4(a)).

For τ_1_ values at 800 nm, the larger excess energy and the larger distance of the electron energies from the Fermi level is reflected in a shorter decay time that can be seen for lower intensities. In our simulations, these values decrease slightly as the intensity increases due to the slight temperature dependence of the electron–electron scattering rate (see Supporting Information Figure S4(c) on the temperature dependence). Surprisingly, according to the measurement, the τ_1_ values get larger at higher intensities. This behavior can not be reproduced by the 3TM. The limitation here might be that the 3TM deals with linear absorption processes, but considering the intensity regimes of the experiments, nonlinear effects should also be considered. For instance, at high enough intensities, electron emission may play a role, diminishing the amount of hot electrons generated, and causing deviation from model predictions. In separate experiments, the photoemission yield was estimated, and it was found to be negligible under our experimental conditions (see Supporting Information Section III regarding the photoemission measurements). Considering a different aspect, it is important to note that for gold, the dominant absorption mechanism is of the interband type. For 800 nm, this means that at larger intensities, multiphoton processes can become more favorable. In this case, the simultaneous absorption of more photons leads to an interband transition. The excess energy in this scenario will be smaller compared to the intraband case, resulting in longer electron scattering time in agreement with the increase in the τ_1_ parameter at 800 nm.

For the τ_2_ parameter of the thermalized electrons, the values and the tendencies are very similar for the two modulation wavelengths (dashed curves in Figure 4(a) and (b)). Although the excitation channels for the electrons are different for the two cases, as soon as the electrons promoted above the Fermi level get thermalized, the energy exchange with the lattice will happen similarly. By increasing the intensity, the electron system reaches higher and higher temperatures for both cases, which simply means longer decay times as observed in the experiments.

We have demonstrated plasmonic probing of a metal-dielectric interface with ∼15 nm interface selectivity and ∼40 fs temporal resolution at the same time. With infrared and blue pump pulses, we could excite intraband and interband transitions at the surface and found that the largest modulation occurs due to the presence of nonthermal electrons, the signal of which is clearly detectable with our current method. The sensitivity and temporal resolution enabled us to measure the very short transient drop in the transmission signal. This corresponds well to the lifetime of nonthermal electrons in our three-temperature model, in which we took into account the accessible energy levels of the electrons excited with the two pump wavelengths and the related temperature dependent scattering rates.

Since we also observed high modulation of the plasmonic signal induced by the free-space beam especially for the intraband excitation, this experimental scheme can also be seen as a prototype of an ultrafast plasmonic switch with switching times on the order of ∼50 fs. Based on physical understanding of the underlying mechanisms as well as modulation depths being close to practical applications, our results have the potential to foster the construction of integrated nanooptical circuitry. In addition, our novel tool enables the investigation of hot electron processes in specific photocatalytic schemes with sizes well below the diffraction limit, which is not possible with the current methods based on transient reflection. As such, this SPP-probe method will stimulate the development of highly efficient surface configurations for these applications also including processes in nanocircuits.

## Supporting Information Available

Measurement scheme, temporal and spectral aspects of the plasmon propagation, and three-temperature model, electron emission measurements (PDF)
