# Supplementary material for: Ultrafast Surface Plasmon Probing of Interband and Intraband Hot Electron Excitations
Source: Nano Lett. 2024 Jun 4;24(26):8024–9. doi: 10.1021/acs.nanolett.4c01669 (PMC11229057; doi:10.1021/acs.nanolett.4c01669)
Supplement: Supplementary file 1 — nl4c01669_si_001.pdf [file nl4c01669_si_001.pdf]

Supporting Information for

Ultrafast surface plasmon probing of interband and intraband hot electron electron excitations

Péter Sándor[╢,#], *Béla Lovász*[╢,#], *Judit Budai*[╦], *Zsuzsanna Pápa*[╢,╦] *and Péter Dombi*[╢,╦,*]

[╢]HUN-REN Wigner Research Centre for Physics, 1121 Budapest, Hungary

[╦]ELI-ALPS Research Institute, 6728 Szeged, Hungary

#L.B. and P.S contributed equally to this work.
* E-mail: dombi.peter@wigner.hun-ren.hu

Section I: Temporal and spectral aspects of the plasmon propagation

The light transmission is measured by taking two spectra at each delay, one with the pump pulse present and one without, integrating the yield separately and then dividing them. The polarization of the pulses are orthogonal to each other, with the plasmon generation pulse polarization lying parallel to the plasmon propagation direction (perpendicular to the incoupler grating grooves, fig. S1(a)).

For the investigation of the non-thermal electrons created with interband excitations, the laser source was a regenerative amplifier system operating with a repetition rate of 1 kHz (Spectra Physics Solstice Ace) used together with an optical parametric amplifier (OPA; Light Conversion TOPAS Prime). The plasmonic probe wavepacket generation and the pump pulses were both derived from the OPA (see figure S1(b)): the latter was set to have a wavelength of 480 nm and pulse duration of 96 fs; the former is the residual pump with 800 nm and about 38 fs duration. Focused spot areas for the pump beam vary between 90-150 μm^2, where the beam size is determined by the top hat criterion[1].

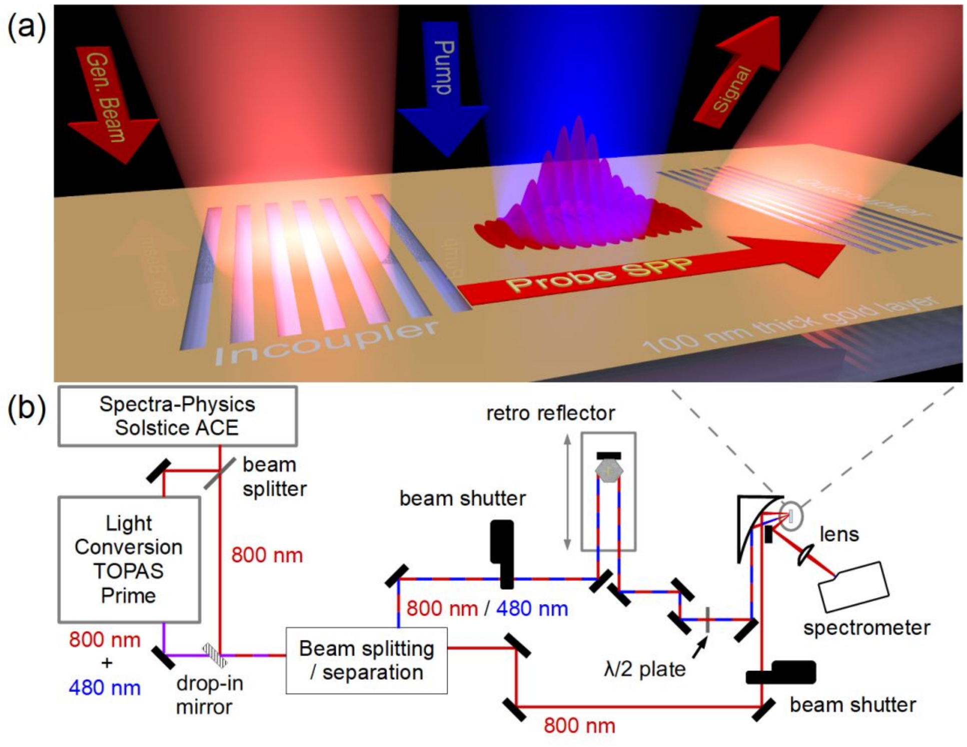

FIG. S1. (a) Illustration of the pump-probe concept with the generation and outcoupling of SPPs using grating couplers milled into the 100-nm gold layer. Nonthermal electron distribution is generated in between the couplers by a separate, time-delayed pump pulse (shown in blue color); (b) Optical layout of the experiment.

Probing the hot electrons created through intraband excitation was carried out by taking the output pulse from the amplifier system directly, splitting it in two and adding variable delay to one of the optical paths. These pulses both have a wavelength of 800 nm and pulse duration of 38 fs. Because of the identical wavelengths, light scattering of the modulation pulse into the detector and the consequent signal pollution and spectral interference at short time delays became an issue. This is why at each delay, an additional spectrum needed to be acquired, this one with only the modulation pulse illuminating the sample, to be subtracted as a background. Despite these extra measures however, the larger noise for the measurements in case of 800 nm pump could not be fully eliminated.

The pulse durations are measured with commercial autocorrelators (APE PulseCheck for 480 nm pulses and APE Mini for 800 nm). For accurate beam positioning, the sample containing the in- and outcoupler, and the portion of the light that was transmitted through the grating grooves, were imaged with a microscope-camera (magnification: 20x – 200x). Characterization of the focal spot size (typically having a diameter of 10-24 μm along the plasmon propagation direction and 6-10 μm perpendicular to it) was done using an *in-situ* knife-edge technique.

The plasmon incoupler and outcoupler gratings have grating constants of a = 800 nm and a = 500 nm respectively. That means that light pulses are coupled into plasmons with an angle of incidence of 0° and light pulses, coupled out from plasmons, leave the sample under an angle of ~ -35°. It has been already demonstrated that gratings can support broadband laser spectra to launch ultrashort plasmon wavepackets with pulse durations even in the range of 10 fs [2].To check that the applied coupler with 800 nm grating constant enables us an efficient coupling by preserving the

temporal and spectral properties of the generating laser pulses, we tested the performance of our incoupler with three-dimensional finite-difference time domain simulations (Lumerical FDTD). We fed the spectrum of the applied source into the software and used the corresponding temporal shape to illuminate the grating structure that has the same geometrical properties as in the experiment. The dielectric function of the 100 nm gold layer was described by the dataset of [3]. Due to the large required computational volume, an adaptive grid was used with a size of 15 nm along the gold-air interface.

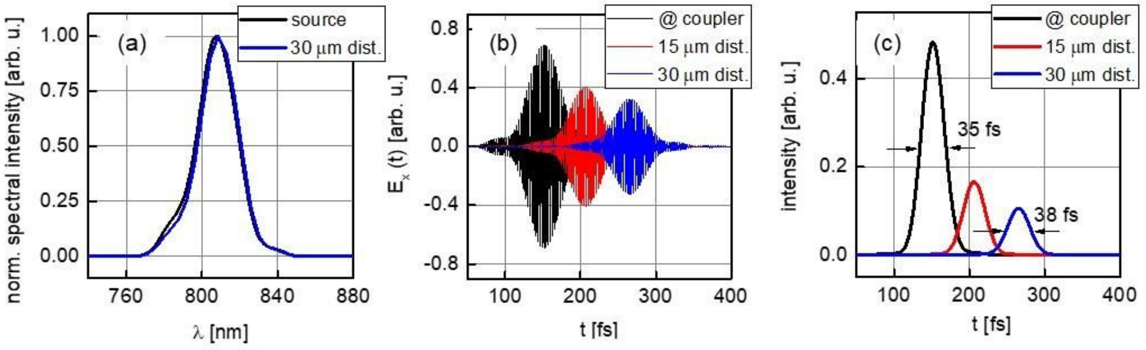

FIG. S2. (a) Spectrum of the incoming pulse and the SPP wave after 30 μm propagation. (b) Time evolution of the incoupled SPP signal upon propagation. (c) The intensity envelopes of the SPP signals upon propagation for analyzing the temporal broadening.

First, we compared the spectrum of the applied source with the spectrum of the SPP signal after 30 μm propagation (Fig. S2(a)). The spectrum is only slightly distorted proving that the applied coupler effectively supports the bandwidth of the short pulse excitation. Another factor that can limit temporal resolution is the chirp during propagation. Since the spectral bandwidth is moderate (<80 nm) in our case, and the propagation length is short (30 μm), these effects can be neglected and the temporal resolution provided by the probe will remain the same during the propagation, as proved by the extracted time signals from FDTD (Fig. S2(b)). Analysis of the intensity FWHM values proved that the incoming 35 fs pulse (transformation limited signal of the corresponding spectrum) is broadened by less than 10% upon SPP coupling and subsequent propagation of the plasmonic wavepacket (Fig. S2(c)).

As a next step, we also checked whether the propagation through the excited area distorts the SPP signal. For this, we analyzed the effect of the laser excitation on the dielectric function of gold. For both applied wavelengths, the contributions of the nonthermal and thermal electron populations to the dielectric function have to be taken into account. According to the results of [4], both contributions cause a comparable increase in the imaginary part of the dielectric function, while the real part changes only slightly. Therefore, we modeled the effect of the thermal electrons where tabulated datasets are available[5]. Since the maximal electron temperature are T_e=4636 K and T_e=4803 K for 480 nm and 800 nm excitations based on our three-temperature model calculations (detailed in the next section), respectively, we used the dielectric function dataset of gold belonging to T_e=5000 K electron temperature and calculated again the spectral and temporal properties of the SPP signal upon propagation through the excited area (Fig. S3).

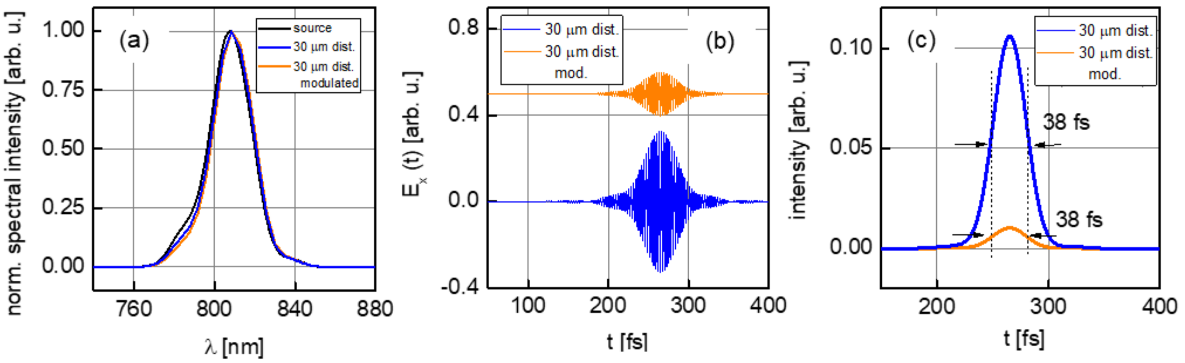

FIG. S3. (a) Spectrum of the incoming pulse and the SPP wave after 30 µm propagation through unexcited and excited/modulated gold. (b) Time signal and (c) intensity envelopes of the incoupled SPP upon propagation through unexcited and excited gold.

Analyzing the spectrum, it is clearly visible that the propagation through the excited part of the gold layer does not affect the spectral content of the SPP pulse. At the same time, the SPP preserves its temporal length as well (Fig. S3(c)). The only difference between the properties of the SPP pulses traveling through the unexcited or the excited gold is in their amplitude: due to the increased imaginary part of the dielectric function in the case of elevated electron temperatures, the losses are larger and the amplitude of the SPP signal drops. Since these increased losses are present also for the contribution of nonthermal electrons[4], the overall transmission of the system will be reduced in both cases explaining the experimentally observed signal drop.

Section II: Three-temperature model

To describe the dynamics of the energy transfer after the ultrafast photoexcitation, three coupled partial differential equations were solved in COMSOL Multiphysics[4]. These equations contain the time and depth dependence of the energy density stored in the population of nonthermal electrons (N), the temperature of the thermal electrons (T_e) and the temperature of the lattice (T_l):

$$\frac{\partial N(x,t)}{\partial t} = -aN - bN + P(x,t), \tag{B1}$$

$$C_e\frac{\partial T_e(x,t)}{\partial t} = -g(T_e - T_l) + aN + \frac{\partial}{\partial x}\left(\frac{T_e}{T_l}\kappa\frac{\partial T_e}{\partial x}\right), \tag{B2}$$

$$C_l\frac{\partial T_l(x,t)}{\partial t} = \kappa\frac{\partial^2 T_l}{\partial x^2} + g(T_e - T_l) + bN. \tag{B3}$$

The source term in equation (B1) is

$$P(x,t) = (1-R)I(t)e^{-x/(\lambda_{ball}+1/\alpha)}/[(\frac{1}{\alpha}+\lambda_{ball})(1-e^{-d/(\lambda_{ball}+1/\alpha)})]$$

containing the absorption coefficient (α), the reflectivity of gold (R), the time dependent intensity of the modulation pulse and the contribution of the ballistic electrons within the ballistic range (λ_{ball}). For gold, a range of $\lambda_{ball} = 100\ nm$ was derived from fs time-resolved measurements, in good agreement with the calculated equilibrium value of the mean free path of electrons[6]. To distinguish between the different modulation wavelengths, the absorption coefficient and the reflectivity were set differently: α= 7.85·10^5 [1/cm] and 4.49·10^5 [1/cm] and R=98% and 37% for 800 nm and 480nm, respectively. With these, one can take into account that immediately after

excitation, when the electrons are in a highly nonequilibrium state, two competing processes take place. One is ballistic motion of these excited electrons into deeper parts of the sample with velocities close to the Fermi velocity, i.e., approximately 10^6 m/s. The other more general process is the development of an electron temperature by collisions between excited electrons and electrons around the Fermi level[6]. This second process is taken into account by the aN term, where a is the electron gas heating rate, being equal to the reciprocal of the electron scattering time and showing dependence both on the electron temperature and electron energy levels:

$$a = \tau_{ee}^{-1} = K\left[(\pi k_B T_e)^2 + (E - E_F)^2\right].$$

In our simulations, we used $K = 8 \times 10^{12} \frac{1}{eV^2 s}$, which is consistent with the values of the literature[7–9]. Since the step-like behavior of the nonequilibrium electron distribution immediately starts to reconfigure after excitation, the most energetic electrons move to energy levels near the half of the maximal electron energy available[10] (see the arrows in Fig. S4 (a) and (b)). Therefore, we considered 0.8 eV excess energy for intraband, and 0.4 eV for interband excitation. With these different energies, the electron-electron scattering time shows different behavior as a function of electron temperature (Fig. S4 (c)).

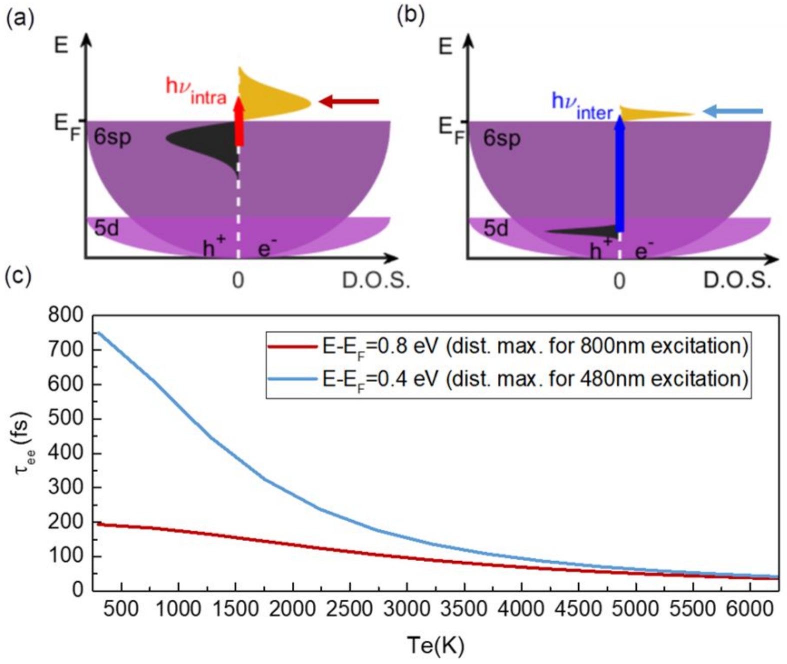

FIG. S4. Schematic representation of the available electron energies for a) intraband and b) interband excitations. Arrows show the center of the distributions giving the bulk of the detected signal. c) Electron scattering time as a function of electron temperature considering different excess energies for the different excitations: red curve represents the intraband excitation with 0.8 eV excess energy, and blue curve shows the interband excitation case.

Some of the energy from the nonthermal electrons can directly heat the lattice. This is described by bN term, where $b = \tau_{el}^{-1} = \frac{k_B T_D}{\tau_f \hbar\omega}$, with T_D =165 K being the Debye temperature and τ_f=13.824 fs is the quasi particle free flight time[4].

C_e is the temperature dependent heat capacity of the electron system, $C_e(T_e) = \gamma T_e$, where $\gamma = 62.9 \frac{J}{m^3 K^2}$ [11], $C_l = 2.5 \times 10^6 \frac{J}{m^3 K}$ is the lattice heat capacity[6], and the electron-phonon coupling constant is denoted with g [11]. The diffusive transport of phonons and thermalized electrons is taken into account with the thermal conductivity of the metal, κ, and that of the electron population, $\kappa_e = \frac{T_e}{T_l}\kappa$, where $\kappa = 318 \frac{W}{mK}$ [12].

Section III: Electron emission measurements

With regards to interpreting the measurements, it has been a concern whether all the possible mechanisms are considered through which the deposited laser energy can leave the system. In particular, ultrashort pulses can not only excite and create hot electrons in the conduction band, but in the appropriate intensity range they can also promote electrons to the continuum. To check the contribution of this phenomenon, we performed separate experiments, in which we illuminated a 100 nm gold film with ultrashort pulses at 480 and 800 nm, varying the incident intensity and recording the number of electrons emitted directly with an MCP, phosphor screen and a camera. The 480 nm pulse had similar duration as in case of the modulation experiments (95 fs), while the 800 nm pulse was 45% longer (55 fs). The laser beams were incident at approx. 40 degrees to the surface of the sample, with s-polarization. The intensity was calibrated by determining the damage threshold, and using that as a basis for comparison with the plasmon modulation experiments (both experiments were carried out using the same sample). Of course, the interaction region was housed in a vacuum chamber with a base pressure of 1e-7 mBar.

Figure S5 shows the results. Datapoints for 480 nm and 800 nm are plotted with light blue and red colors, respectively, along with their respective damage thresholds (dashed vertical lines). One can see that just below the damage threshold, at 800 nm the electron yield is more than a magnitude larger than at 480 nm; however needless to say that the intensity is also a lot higher. The puzzling aspect of these measurements is the fact that in principle when s-polarization is used, the electric field has no normal component to the sample surface and hence no emission is expected. In practice however, we found that the polarization contrast of the laser is not perfect; in fact, it's 100:1, and we attribute the observed emission to the residual p-polarization.

We would like to stress that the goal of these experiments was to give an order-of-magnitude estimate of the yield, and they are in no way meant to be interpreted as exacting measurements. For instance, at the higher intensities for 800 nm wavelength, some degree of detector saturation cannot be ruled out. Also, in the actual modulation experiment, the incident angle of the modulation beams were about half of what was used here, most likely leading to less emission at the same intensity. Having said these, the results show that at both wavelengths and for all the used intensities, the yield stays at or below an electron per laser pulse per μm^2. Our conclusion is that this amount of emission is marginal.

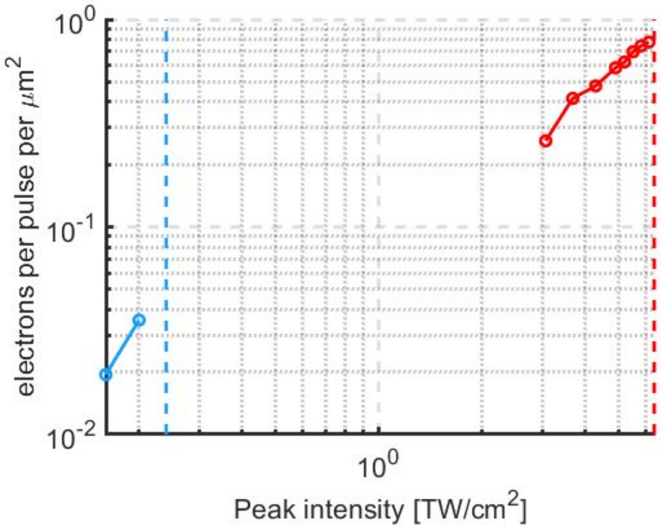

FIG. S5. Electron emission yield from a 100 nm thick gold layer, as a function of incident laser peak intensity, at wavelengths of 480 nm and 800 nm (solid light blue and red curves, respectively). Dashed lines show the corresponding damage thresholds.

REFERENCES

(1) Siegman, A. E. *Lasers*; University Science Books, 1986.

(2) Schmidt, S.; Piglosiewicz, B.; Sadiq, D.; Shirdel, J.; Lee, J. S.; Vasa, P.; Park, N.; Kim, D.-S.; Lienau, C. Adiabatic Nanofocusing on Ultrasmooth Single-Crystalline Gold Tapers Creates a 10-Nm-Sized Light Source with Few-Cycle Time Resolution. *ACS Nano* **2012**, *6* (7), 6040–6048. https://doi.org/10.1021/nn301121h.

(3) Haynes, W. M. *CRC Handbook of Chemistry and Physics, 96th Edition*; CRC Press, 2015.

(4) Schirato, A.; Maiuri, M.; Toma, A.; Fugattini, S.; Proietti Zaccaria, R.; Laporta, P.; Nordlander, P.; Cerullo, G.; Alabastri, A.; Della Valle, G. Transient Optical Symmetry Breaking for Ultrafast Broadband Dichroism in Plasmonic Metasurfaces. *Nat. Photonics* **2020**, *14*, 723–727. https://doi.org/10.1038/s41566-020-00702-w.

(5) Yurkevich, A. A.; Ashitkov, S. I.; Agranat, M. B. Permittivity of Gold with a Strongly Excited Electronic Subsystem. *Phys. Plasmas* **2017**, *24*, 113106. https://doi.org/10.1063/1.5000285.

(6) Hohlfeld, J.; Wellershoff, S.-S.; Güdde, J.; Conrad, U.; Jähnke, V.; Matthias, E. Electron and Lattice Dynamics Following Optical Excitation of Metals. *Chem. Phys.* **2000**, *251* (1), 237–258. https://doi.org/10.1016/S0301-0104(99)00330-4.

(7) Dubi, Y.; Sivan, Y. “Hot” Electrons in Metallic Nanostructures—Non-Thermal Carriers or Heating? *Light Sci. Appl.* **2019**, *8* (1), 89. https://doi.org/10.1038/s41377-019-0199-x.

(8) Carpene, E. Ultrafast Laser Irradiation of Metals: Beyond the Two-Temperature Model. *Phys. Rev. B* **2006**, *74* (2), 024301. https://doi.org/10.1103/PhysRevB.74.024301.

(9) Riffe, D. M.; Wilson, R. B. Excitation and Relaxation of Nonthermal Electron Energy Distributions in Metals with Application to Gold. *Phys. Rev. B* **2023**, *107* (21), 214309. https://doi.org/10.1103/PhysRevB.107.214309.

(10) Della Valle, G.; Conforti, M.; Longhi, S.; Cerullo, G.; Brida, D. Real-Time Optical Mapping of the Dynamics of Nonthermal Electrons in Thin Gold Films. *Phys. Rev. B* **2012**, *86* (15), 155139. https://doi.org/10.1103/PhysRevB.86.155139.
(11) Lin, Z.; Zhigilei, L. V.; Celli, V. Electron-Phonon Coupling and Electron Heat Capacity of Metals under Conditions of Strong Electron-Phonon Nonequilibrium. *Phys. Rev. B* **2008**, *77* (7), 075133. https://doi.org/10.1103/PhysRevB.77.075133.
(12) Qiu, T. Q.; Tien, C. L. Heat Transfer Mechanisms During Short-Pulse Laser Heating of Metals. *J. Heat Transf.* **1993**, *115* (4), 835–841. https://doi.org/10.1115/1.2911377.
